# Effect Analysis of Preoperative Intravenous Tranexamic Acid Combined With Intraoperative Immersion in Reducing Perioperative Blood Loss of One Stage Posterior Thoracolumbar Tuberculosis

**DOI:** 10.3389/fsurg.2022.852589

**Published:** 2022-06-23

**Authors:** Bowen Zheng, Boyv Zheng, Huaqing Niu, Xiaobin Wang, Guohua Lv, Jing Li, Jingyu Wang

**Affiliations:** ^1^ Department of Spine Surgery, The Second Xiangya Hospital of Central South University, Changsha, China; ^2^ Musculoskeletal Tumor Center, Peking University People's Hospital, Peking University, Beijing, China; ^3^ Department of Orthopedics Surgery, General Hospital of the Central Theater Command, Wuhan, China

**Keywords:** thoracolumbar tuberculosis, tranexamic acid, blood loss, local immersion, drainage

## Abstract

**Background:**

To investigate the efficacy and safety of preoperative intravenous tranexamic acid (TXA) combined with intraoperative immersion in reducing perioperative blood loss in one-stage posterior thoracolumbar tuberculosis.

**Methods:**

All patients were divided into four groups: Group A received an intravenous drip of TXA before surgery, group B received multiple local immersions during the operation, group C received an intravenous drip combined with multiple local immersions, and the control group (group CG) were not treated with TXA during the same period. The total blood loss (TBL), intraoperative blood loss (IBL), hidden blood loss (HBL), postoperative drainage volume, maximum hemoglobin drop value (max Hb drop), liver and kidney function, coagulation indexes, blood transfusion rate, hospital stay and incidence of complications were compared among the four groups.

**Results:**

TBL, IBL, HBL, max Hb drop, POD1 drainage, and POD2 drainage in group A, group B, and group C were significantly lower than those in group CG. TBL, IBL, HBL and max Hb drop were group C < group A < group B < group CG. The drainage volume of group C was significantly lower than that of the other groups. There was no significant difference in blood coagulation index (PT, D-D) or liver and kidney function (ALT, Cr) among the four groups. There was no difference in postoperative hospital stay between group A and group B, but it was significantly lower in group C than in the other three groups. All patients achieved satisfactory bone graft fusion at the last follow-up.

**Conclusion:**

Preoperative intravenous drip of TXA combined with intraoperative multiple immersion can effectively reduce perioperative blood loss while not increasing the risk of thrombosis without affecting liver and kidney function, coagulation function or tuberculosis prognosis.

## Introduction

Typical thoracolumbar tuberculosis (T11-L2) is characterized by intervertebral disc involvement, destruction of adjacent vertebrae and formation of paraspinal abscesses, which usually require surgical treatment. Compared with other approaches, one-stage posterior focus debridement, bone grafting and pedicle screw internal fixation have the advantages of less trauma, faster recovery, stable internal fixation and better effect of deformity correction, which is accepted by the majority of spinal surgeons (1, 2). In spite of this, tuberculosis surgery itself still has the problems of long operation time and large blood loss, which brings great challenges to the safety of operation, and most tuberculosis patients are accompanied by anemia and hypoproteinemia due to long-term chronic nutritional consumption. For patients with poor physique, perioperative blood loss is not conducive to postoperative rehabilitation. Blood transfusion not only increases medical expenses but also may cause complications such as disease transmission, hemolysis, postoperative epidural hematoma, and allergic reaction (3, 4). Therefore, it is particularly important to control perioperative blood loss in patients with spinal tuberculosis.

In addition to improving surgical skills and shortening the time of operation to reduce bleeding, the rational use of hemostatic drugs is also a good choice. Tranexamic acid (TXA) is a synthetic lysine derivative. It binds to the lysine binding site of plasmin/plasminogen and then inhibits the fibrinolysis mediated by plasmin to achieve hemostasis (5). It was first used in clinical practice in the 1960s, and most studies have proven that it can effectively reduce perioperative blood loss and the blood transfusion rate in cardiology, obstetrics, urology, orthopedics and other specialties (6–9). The route of TXA is mainly divided into intravenous and topical routes. The effectiveness and safety of intravenous, intra-articular or combined medication for reducing perioperative blood loss in knee arthroplasty has been confirmed (10, 11). There are many studies about intravenous TXA in spinal surgery, and it is basically agreed that TXA can reduce blood loss and hospital stay (12). However, there are few reports of topical TXA, and its effect is still controversial (13, 14).

To the best of our knowledge, there are no reports about the application of TXA in one-stage posterior thoracolumbar tuberculosis surgery. We achieved a good hemostatic effect by using a preoperative intravenous drip combined with multiple immersions during the operation. The purpose of this study was to evaluate the efficacy and safety of intravenous, topical and combined TXA administration in reducing perioperative blood loss in thoracolumbar tuberculosis and to provide a reference for clinical treatment.

## Materials and Methods

### Patients

This study was approved by the Ethics Committee of The Second Xiangya Hospital of Central South University, and all the patients signed the informed consent form before the operation. Patients who underwent one-stage posterior focus debridement, interbody fusion and pedicle screw fixation for thoracolumbar tuberculosis from July 2014 to February 2019 were included in the study. The inclusion criteria were as follows: (1) patients with clinical manifestations, laboratory examination and postoperative pathology confirmed as tuberculosis; (2) single segmental thoracolumbar tuberculosis; (3) destruction of the anterior column with or without kyphosis and nerve injury; and (4) poor effect of conservative treatment. The exclusion criteria were as follows: (1) active pulmonary tuberculosis; (2) combined anterior and posterior surgery or conservative treatment; (3) coagulation dysfunction; (4) severe hepatic and renal dysfunction; (5) cardiovascular and cerebrovascular diseases such as myocardial infarction, cerebral infarction, atrial fibrillation, and angina pectoris; and (6) use of anticoagulants and antiplatelet drugs within 7 days before the operation.

**Figure 1:**
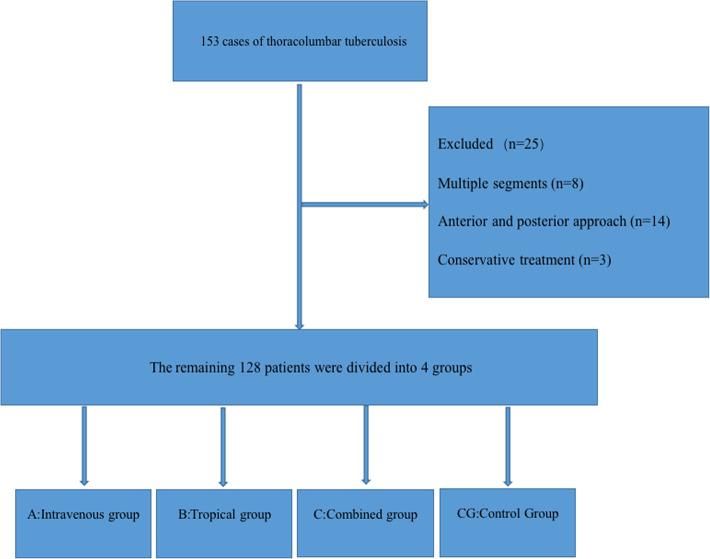
Experimental flow chart.

Eligible patients were divided into four groups: group A (*n* = 32) received 20 mg/kg TXA intravenously 15 min before the operation; group B (*n* = 30) was treated with 300 mL of 3 g TXA saline solution to soak the wound during the operation; group C (*n* = 34) received a preoperative intravenous drip combined with intraoperative multiple immersion; and the control group (group CG, *n* = 32) were not treated with TXA during the same period (Figure 1).

Ninety-six patients using TXA were followed up for more than 18 months, including 55 males and 41 females (mean age 45.43 years, range 27–67 years). There were 22 cases with T10-T11, 24 cases with T11-T12, 21 cases with T12-L1 and 29 cases with L1-L2. The control group included 18 males and 14 females (mean age 47.69 years, range 29–66 years). Of these, 7 were T10-T11, 8 were T11-T12, 7 were T12-L1, and 10 were L1-L2, Similarly, 32 patients in the control group were also followed up for more than 18 months.

### Preoperative Treatment

All patients were treated with four regular antituberculosis drugs (isoniazid 5 mg/kg, rifampicin 10 mg/kg, ethambutol 15 mg/kg, pyrazinamide 25 mg/kg) for 2 to 4 weeks. Surgical treatment was performed after the erythrocyte sedimentation rate, C-reactive protein, body temperature returned to normal or significantly decreased, anemia and hypoproteinemia were corrected, and the general condition improved.

### Operation Method

The operation was performed by the same group of doctors. A posterior midline incision was made, and the paravertebral muscles were dissected under the periosteum to expose the lamina, articular process, transverse process, costal transverse process and medial rib. Pedicle screws were inserted into the two vertebral bodies above and below the affected vertebra, and short screws were placed in the affected vertebra as appropriate. To avoid spinal cord injury caused by spinal instability during the operation, temporary rod fixation was used on the side with mild lesions. A small piece of rib (approximately 2 cm) and part of the facet joint were removed on the severe side, and then the pleura was carefully pushed outward. The intercostal nerves on one side could be sacrificed as needed to fully expose the lesions. The dead bone, tuberculous granulation tissue and pus were removed under direct vision. Then, a prebending rod was used to correct kyphosis, and the trimmed ribs or ilium was implanted into the intervertebral body. After proper compression, the screw tail cap was locked, and a posterolateral bone graft was performed. The gelatin sponge containing 0.4 g isoniazid and 0.8 g amikacin was placed into the focus. The drainage tube was placed, and the incision was sutured layer by layer.

### Usage of TXA

Patients in group A were given 100 mL of 20 mg/kg TXA saline solution intravenously before the operation, and 100 mL saline was used for local irrigation after paravertebral muscle dissection, after lesion exposure and before incision closure. In group B, 100 mL saline was intravenously dripped 15 min before the operation. Meanwhile, 300 mL of 3 g TXA saline solution was divided into three equal parts, local immersion was performed after paravertebral muscle dissection, after lesion exposure and before incision closure for 2 min, 2 min, 5 min, and then sucked away; group C received intravenous drip of 100 mL of 20 mg/kg TXA saline 15 min before operation, and 100 mL of 1 g TXA saline solution was used to soak the wound after paravertebral muscle dissection, after lesion exposure and before incision closure, respectively.

### Postoperative Management and Evaluation Index

After the operation, patients were given air pressure pump treatment of both lower extremities, and patients were encouraged to perform isometric contractile exercise of lower limb muscles to prevent deep venous thrombosis (DVT)). The drainage tube was removed when the drainage volume was less than 30 mL/d. Antibiotics were used for 5–7 days, and the treatment of four antituberculosis drugs was continued. Pyrazinamide was stopped after 3 months, and the other three antituberculosis drugs lasted for 12–18 months. The patient can wear a brace and leave the bed for appropriate activities after 2 weeks, but bed rest should still be the mainstay before bone graft fusion. All patients were followed up at 1, 3, 6, 12, and 18 months after surgery and then reviewed once a year.

The operation time, postoperative hospital stay, hemoglobin (Hb), hematocrit (HCT), D-dimer (D-D), prothrombin time (PT), glutamic pyruvic transaminase (ALT) and creatinine (Cr) were recorded before and after the operation.

The total blood loss (TBL) was calculated according to the estimated blood volume (EBV) formula of Nadler et al. (15). TBL = 2*EBV (Hctpre-Hctpost)/(Hctpre + Hctpost), Hctpre was Hct on the day before the operation, and Hctpost was Hct on the third day after the operation. Visible blood loss (VBL) = intraoperative blood loss (IBL) + postoperative drainage, hidden blood loss (HBL) = TBL-VBL. If the patient received a blood transfusion during the period, HBL = TBL+ transfusion volume-VBL. The maximum hemoglobin drop value (Max Hb drop) = Hbpre-Hblowest (Hbpre is the level of Hb one day before operation; Hblowest is the lowest value within 3 days after operation).

Routine ultrasound examination of the lower extremities was performed before discharge or at any time when the patient had lower limb pain and swelling to determine whether there was deep venous thrombosis (DVT). Perioperative complications, such as incision infection, epidural hematoma, epilepsy, DVT, pulmonary embolism, visual impairment, myocardial ischemia and ischemic encephalopathy and final bone graft fusion, were recorded.

### Statistical Processing

Spss23.0 was used to analyze the data. Continuous variables were expressed as the mean ± SD. The data of four groups were compared by one-way ANOVA. When there was a significant difference among the four groups, the Bonferroni test was used for pairwise comparisons. The chi-square test was used to compare categorical variables. When *P* < 0.05, the difference was considered statistically significant.

## Results

All patients were followed up for at least 18 months, and satisfactory implant fusion was achieved at the last follow-up. There were no significant differences between the four groups in terms of age, sex, BMI, lesion segment, time to surgery, or other baseline data (Table 1).

**Table 1:** Baseline parameters of patients in the 4 groups.

Variable	Group A	Group B	Group C	Control Group	*P*-value
M/F	20/12	16/14	19/15	18/14	0.750
Age (years)	45.24 ± 9.85	45.44 ± 9.58	45.61 ± 9.65	45.43 ± 9.72	0.597
BMI (kg/cm^2^)	20.06 ± 1.67	20.12 ± 1.59	20.14 ± 1.44	20.09 ± 1.67	0.645
Lesion location (*n*)					0.965
T10∼T11	7	6	9	7	
T11∼T12	7	9	8	8	
T12∼L1	8	7	6	7	
L1∼L2	10	8	11	10	
Preopertive Hb	130.31 ± 10.38	130.49 ± 10.80	129.96 ± 10.32	130.36 ± 10.78	0.069
Operative time (min)	244.15 ± 26.10	244.08 ± 24.52	243.57 ± 25.70	243.95 ± 25.91	0.291
Incision length (cm)	9.62 ± 1.49	9.74 ± 1.73	9.87 ± 1.60	9.77 ± 1.69	0.149

*M, male; F, female; BMI, body mass index; Hb, hemoglobin.*

TBL, IBL, HBL, max Hb drop, POD1 drainage, and POD2 drainage in group A, group B, and group C were significantly lower than those in group CG. TBL, IBL and HBL in group C were significantly lower than those in groups A and B, while group A was significantly lower than group B. There was no significant difference in preoperative Hb between the four groups, and the max Hb drop after surgery was (group C < group A < group B < group CG). On the first postoperative day, the drainage in group C was significantly lower than that in group A, group B and group CG, while group A was lower than group B and group CG, and group CG had the most drainage; on the second day, there was no significant difference between group A and group B, while group C was still lower than the other three groups. On the third day, there was no significant difference between the four groups. The transfusion rates of the four groups were 18.75%, 16.67%, 5.88% and 15.63%, respectively, but were not statistically significant. There was no difference in the postoperative hospitalization time between groups A and B, but the hospitalization time in group CG was significantly higher than that in the other groups, and the hospitalization time in group C was the shortest (Table 2).

**Table 2:** Postoperative outcome among the 4 groups.

Variable	Group A	Group B	Group C	Control Group	*P* value	*P* AvsB	*P* AvsC	*P* BvsC	*P* CGvsA	*P* CGvsB	*P* CGvsC
Max Hb drop (g/L)	28.06 ± 6.73	31.73 ± 5.57	22.65 ± 5.13	55.31 ± 10.39	<0.001	0.046	0.001	<0.001	<0.001	<0.001	<0.001
TBL (mL)	1047.08 ± 111.97	1146.99 ± 137.29	878.04 ± 103.04	1411.06 ± 153.67	<0.001	<0.001	<0.001	<0.001	<0.001	<0.001	<0.001
IBL (mL)	365.52 ± 73.71	405.52 ± 67.18	318.38 ± 71.43	512.73 ± 79.79	<0.001	<0.001	<0.001	<0.001	<0.001	<0.001	<0.001
HBL (mL)	432.16 ± 80.02	461.28 ± 63.49	384.07 ± 56.04	497.63 ± 91.32	<0.001	<0.001	<0.001	<0.001	<0.001	<0.001	<0.001
POD1 drainage (mL)	161.95 ± 31.69	186.46 ± 38.70	123.70 ± 30.64	201.39 ± 51.68	<0.001	<0.001	<0.001	<0.001	<0.001	<0.001	<0.001
POD2 drainage (mL)	95.85 ± 15.31	96.66 ± 14.15	59.33 ± 19.13	102.33 ± 25.61	<0.001	0.159	<0.001	<0.001	<0.001	<0.001	<0.001
POD3 drainage (mL)	35.59 ± 10.84	35.11 ± 9.93	34.81 ± 11.66	35.44 ± 11.22	0.258	/	/	/	/	/	/
Transfusion (case)	6	5	2	5	0.235	/	/	/	/	/	/
Post-op HOS (days)	13.75 ± 2.08	13.55 ± 1.81	12.02 ± 1.75	14.07 ± 1.98	<0.001	0.387	<0.001	<0.001	<0.001	<0.001	<0.001
Wound infection (case)	2	1	3	2	0.869	/	/	/	/	/	/
DVT (case)	0	1	0	1	0.312	/	/	/	/	/	/

*Max Hb drop, the maximum hemoglobin drop value; TBL, total blood loss; IBL, intraoperative blood loss; HBL, hidden blood loss; POD1-3, post-operative day 1-3; Post-op HOS, postoperative hospital stay; DVT, deep venous thrombosis.*

There was no significant difference in the coagulation indexes (PT, D-D) before and 3 days after surgery, and there was no significant difference in the liver and kidney function indexes (ALT, Cr) before and 3 days after surgery among the four groups (Table 3).

**Table 3:** Preoperative and postoperative blood biochemical indexes among the 4 groups.

Varible	Group A	Group B	Group C	Control Group	*P* value
Pre-op D-D (μg/mL)	0.48 ± 0.23	0.64 ± 0.36	0.58 ± 0.34	0.55 ± 0.33	0.490
POD3 D-D (μg/mL)	1.39 ± 0.35	1.48 ± 0.39	1.30 ± 0.37	1.49 ± 0.36	0.158
Pre-op PT (s)	12.11 ± 1.36	12.80 ± 1.04	12.60 ± 1.12	12.51 ± 1.09	0.065
POD3 PT (s)	12.97 ± 1.13	13.59 ± 1.04	13.11 ± 1.11	13.37 ± 1.03	0.060
Pre-op ALT (U/L)	21.38 ± 8.86	22.70 ± 8.08	19.74 ± 7.44	21.19 ± 7.96	0.348
POD3 ALT (U/L)	24.62 ± 9.40	25.11 ± 8.12	24.32 ± 9.31	25.02 ± 8.49	0.274
Pre-op Cr (μmol/L)	75.26 ± 13.70	77.33 ± 11.21	76.98 ± 12.16	76.86 ± 12.03	0.778
POD3 Cr (μmol/L)	78.58 ± 12.78	81.47 ± 11.86	77.84 ± 10.60	79.85 ± 11.66	0.439

*D-D, D dimer; PT, prothrombin time; ALT, Alanine transaminase; Cr, Creatinine; Pre-OP, pre-operation; POD 3, post-operative day 3.*

No serious adverse events, such as pulmonary embolism, epidural hematoma, epilepsy, myocardial ischemia, visual impairment or ischemic encephalopathy, occurred in any patient. Only one patient in each of the B and CG groups developed slight swelling of the lower extremities at 5 and 7 days postoperatively. B-ultrasound showed intermuscular venous thrombosis, and the symptoms improved with active treatment. One patient in group A had sinus tract formation, which was cured with local debridement and intensive dressing changes. In addition, two case in group A, one case in group B, three cases in group C, and two cases in group CG had superficial infection of the incision, which was cured after anti-infection, drug change and other symptomatic treatment.

## Discussion

The local blood supply of the spine is abundant, and exposure of the operative area and osteotomy will lead to hyperfibrinolysis and increased blood loss (16). Compared with combined anterior and posterior surgery, one-stage posterior tuberculosis focus debridement and bone graft internal fixation shorten the operation time and reduce the amount of trauma and blood loss to a certain extent, but its exposure range and visual field are limited, which is not conducive to hemostasis during the operation. The exposed bone surface is not suitable for standard hemostasis in soft tissue surgery, and the deeper wound cannot be effectively oppressed, resulting in large postoperative drainage (17). Tuberculosis patients are prone to malnutrition, anemia and hypoproteinemia due to long-term poor appetite and lack of sleep, so ensuring a good systemic condition is an important part of anti-tuberculosis treatment (18). Perioperative blood loss will lead to worse immunity in tuberculosis patients and prolong the time of hospitalization and rehabilitation. The successful application of TXA in other orthopedic surgeries provides us with a good reference. We used different methods of TXA for perioperative hemostasis of spinal tuberculosis and achieved satisfactory results in long-term follow-up.

TXA can effectively reduce bleeding by inhibiting fibrinolysis and stabilizing blood clots. A number of studies have shown that intravenous administration of the antifibrinolytic drug TXA during spinal surgery can significantly reduce blood loss, blood transfusion volume and blood transfusion rate; however, there is no consensus on the optimal timing and dosage of intravenous administration (19, 20). In view of the fact that the half-life of single-dose intravenous administration is 120 min, it is generally reported that dual-dose or preoperative load dose supplemented by maintenance dose. Raksakietisak et al. (21) used 15 mg/kg TXA intravenously at the beginning of 39 cases of complex spinal surgery and 3 h later, which resulted in lower blood loss and transfusion rates than the placebo group. Elwatidy et al. (22) used 2 g TXA intravenously before spinal surgery in 64 cases, and supplemented with a 100 mg/h maintenance dose within 5 h after the operation, the blood loss was 49% less than that of the placebo group. Raman et al. (16) considered that the hemostatic effect of a high dose (load 30 mg/kg, maintenance dose 1–10 mg/kg/h) was better than that of a low dose (load dose 10 mg/kg, maintenance dose 1–2 mg/kg/h) in adult spinal deformities. However, the incidence of postoperative atrial fibrillation and myocardial infarction was higher in the high-dose group.

A high load dose, additional maintenance dose or repeated use of TXA can significantly inhibit fibrinolytic activity, and the imbalance of the fibrinolytic system is closely related to thrombosis (23). Systemic administration of TXA can penetrate the blood–brain barrier and then spread throughout the central nervous system (5). Although complications of intravenous TXA are rare, they do exist, especially in patients with hypercoagulability, severe renal failure and ischemic heart disease (24). High-dose intravenous administration of TXA in patients undergoing cardiac surgery has been reported to lead to nonischemic seizures (25). On the other hand, single-dose application also achieved good results. Sun et al. (26) intravenously dripped 15 mg/kg TXA 30 min before lumbar fusion surgery, and IBL and drainage volume 24 h after surgery were significantly reduced compared with the placebo group. Wang (27) et al. used 15 mg/kg TXA intravenously 15 min before lumbar degeneration surgery, and the postoperative blood loss was 13% less than that of the control group. To reduce the potential complications caused by systemic medication and considering the time-consuming operation of tuberculosis and the ceiling effect of drugs, we chose to intravenously drip 20 mg/kg TXA 15 min before surgery, combined with multiple immersions during the operation, in an attempt to enhance its hemostatic effect. The results are satisfactory.

The application of topical TXA in joint surgery is relatively mature. A multicenter randomized controlled study showed that topical 1–3 g TXA was effective in knee arthroplasty (28). A meta-analysis of topical application of TXA in spine surgery showed that methods such as local soaking or indwelling gelatin sponge with TXA before incision closure can also reduce blood loss and drainage while not increasing the risk of complications (29). Xu et al. (30) found that local immersion with 1 g TXA before closing the incision in spinal fusion surgery significantly reduced postoperative drainage compared with gelatin sponge and collagen hemostatic sponge. Ren et al. (17) used 100 mL of 1 g TXA saline to soak the wound for 5 min before incision closure in 50 patients undergoing lumbar fusion, and the postoperative blood loss was 44.29% of the control group, while shortening the hospital stay. Compared with intravenous medication, this method is convenient to manage and can provide a higher concentration in the bleeding area, inhibiting the fibrinolytic activity of the local tissue and preventing the fibrin clot from dissolving, increasing the volume and strength on the wound surface, thereby enhancing microvascular coagulation while reducing system exposure (31, 32). In theory, it may be better to prolong the soaking time and increase the absorption of drugs by local tissues, but that will prolong the time of operation and anesthesia, and spine surgery cannot prolong drug absorption time by clamping the drainage tube as it does after joint replacement. Therefore, unlike the previously reported single-dose local administration before incision closure, we used TXA solution to soak wounds after paravertebral muscle dissection, after lesion exposure and before incision closure. The results showed that although the hemostatic effect was slightly worse than that of intravenous administration alone, the combination was better than that of single administration.

In this study, the combined group was superior to the single-medication group in reducing TBL, IBL, and HBL, which indicated that intravenous drip before the operation, soaking and flushing during the operation simultaneously not only reduced the total blood loss but also made the surgical field cleaner and increased the safety of the operation. A single intravenous administration could maintain the effective plasma concentration for approximately 16 h (33), and resoaking the wound at the end of the operation further prolonged the action time of the drug, so the postoperative drainage was significantly less than that of the other groups. The blood transfusion rate in the combined group (5.88%) was lower than that in the other two groups (16.67%, 18.75%), but there was no statistical significance in the blood transfusion rate among the three groups. The result may be due to the small sample size on the one hand and the strict grasp of the indication for blood transfusion on the other hand. Less blood loss in the combined group means that the general condition recovers quickly, and early functional exercise makes the length of hospital stay significantly lower than that in the other two groups. TXA has a low price, which is much lower than the cost of blood transfusion and prolonged hospitalization. Therefore, preoperative intravenous drip combined with intraoperative multiple local immersion for hemostasis not only contributes to the recovery of spinal tuberculosis but also reduces their economic pressure.

Due to the antifibrinolytic effect of TXA, most users are concerned about whether it increases the risk of thrombosis, leading to DVT, ischemic cerebral infarction, myocardial infarction and pulmonary embolism. In this study, there was no significant difference in the indexes of coagulation function among the four groups, and there were no serious complications of thrombosis in the perioperative period, which again proved that TXA could inhibit fibrinolysis within the effective drug action window but had no significant effect on coagulation function and did not increase the incidence of thrombosis (34, 35). The reason may be attributed to the fact that the inhibitory effect of TXA on fibrinolysis is mainly located in the surgical wound rather than in the circulatory system, and it has no effect on the vein wall (36). Among them, 1 case of postoperative intermuscular venous thrombosis was considered to be related to his refusal to use a lower limb air pressure pump after the operation. Therefore, for the sake of safety, we believe that postoperative intervention measures such as active use of a lower limb air pressure pump and encouraging patients to carry out isometric contraction of lower limb muscles are of great benefit to the prevention of thrombosis. In addition, the number of incision infections in the four groups was similar, and satisfactory bone graft fusion was achieved in the four groups after long-term follow-up, indicating that the drug would not affect the short-term and long-term prognosis of patients with spinal tuberculosis.

### Limitations

There are some limitations in this study. First, this is a single-center retrospective cohort study. Due to the low incidence of perioperative complications, the small sample size may not be able to identify all complications, e.g. the conclusion that “while not increasing the risk of thrombosis without affecting liver and kidney function, coagulation function or tuberculosis prognosis” may be inaccurate Secondly, the medication method of this study is empirical medication, and the optimal dosage and administration time need to be further explored. In the future, further large-scale prospective experiments are needed to further explore and verify this conclusion.

## Conclusion

Preoperative intravenous drip of TXA combined with intraoperative multiple immersion can effectively reduce perioperative blood loss and shorten hospital stay while not increasing the risk of thrombosis without affecting liver and kidney function, coagulation function or tuberculosis prognosis.

## Data Availability

The original contributions presented in the study are included in the article/Supplementary Material, further inquiries can be directed to the corresponding author/s.
